# Do we really allow patient decision-making in rotator cuff surgery? A prospective randomized study

**DOI:** 10.1186/s13018-019-1157-2

**Published:** 2019-04-29

**Authors:** Carlos Torrens, Joan Miquel, Fernando Santana

**Affiliations:** 1 Department of Orthopedics, Hospital del Mar, Passeitg Marítim 25-29, 08003 Barcelona, Spain; 2 Department of Orthopedics, Hospital d’Igualada, Consorci Sanitari l’Anoia, Barcelona, Spain

**Keywords:** Patient participation, Shared decision-making, Outcomes, Rotator cuff, Rotator cuff-repair

## Abstract

**Background:**

There is a growing patient interest in being involved in the decision-making process. However, little information is provided on how this information should be structured.

Does it make a difference, in patient treatment decision-making, whether information is given based on the benefits or on the side effects in rotator cuff disorders?

**Methods:**

It is a prospective randomized study that includes patients diagnosed with rotator cuff tears.

Patients were randomly allocated to either group A (benefit-inform) or group B (side effect-inform) and were asked to answer the following questions based on their assigned group:

Group A: Your doctor informs you that you have a rotator cuff tear and states that if he/she surgically repairs your cuff tear you will improve and that the cuff remains healed at the 2-year follow-up in 71% of the cases where surgery is done.

Would you choose surgery? Yes or No

Group B: Your doctor informs you that you have a rotator cuff tear and that if he/she surgically repairs your cuff tear you will improve and that the cuff is torn again at 2-year follow-up in 29% of the cases where surgery is done.

Would you choose surgery? Yes or No

Age, gender, the shoulder affected and the functional status assessed through the Constant score were also recorded.

**Results:**

80 patients were randomized (43 to group A and 37 to group B). The patients assigned to group A (benefit) accepted surgery significantly more frequently than those assigned to group B (complication) (*P* = 0.000). In group A, 36 of 43 (84%) accepted surgery, compared to 17 of 37 (46%) in group B.

**Conclusions:**

The way that information on rotator cuff disorders is provided strongly influences patients’ treatment decisions.

**Trial registration:**

ClinicalTrials.gov, NCT03205852. Registered 29 June 2017. Retrospectively registered.

## Introduction

The paternalistic approach to treatment decision-making based on the doctor’s preferences has largely been superseded by involving patients throughout the decision-making process where possible. Moreover, it has been shown that patient involvement results in more cost-effective services and improves health outcomes, quality of life and patient satisfaction [1–7].

Most health conditions can be approached with different strategies that have implicit advantages and disadvantages. Rotator cuff disorders often have various conservative and surgical approaches, each with their own risks and benefits [8]. Many studies have emerged in recent years to determine the optimal degree of involvement for patients, how patients make treatment choices and factors solely related to the physician that affect patient participation [1–3, 6, 7, 9–16].

It seems crucial to provide the patient with information about the pros and cons of different treatment strategies and share treatment decision-making. However, little information is provided on how this information should be structured. Should we give the patient information based on the benefits or should we give the information based on the complications of the treatment?

The objective of this study was to evaluate the effect on patient treatment decision-making if information was given based on the benefit or on the complications in rotator cuff disorders. A secondary objective was to examine the association of socio-demographic variables and functional impairment in the patient decisions.

## Materials and methods

A prospective randomized study was conducted of patients diagnosed with rotator cuff tears who had been recruited at their first clinical visit. The rotator cuff tears were documented with magnetic resonance imaging (MRI) in all patients. Patients were excluded if they had had previous shoulder surgery, worker’s compensation, or were unwilling to participate.

**Fig. 1:**
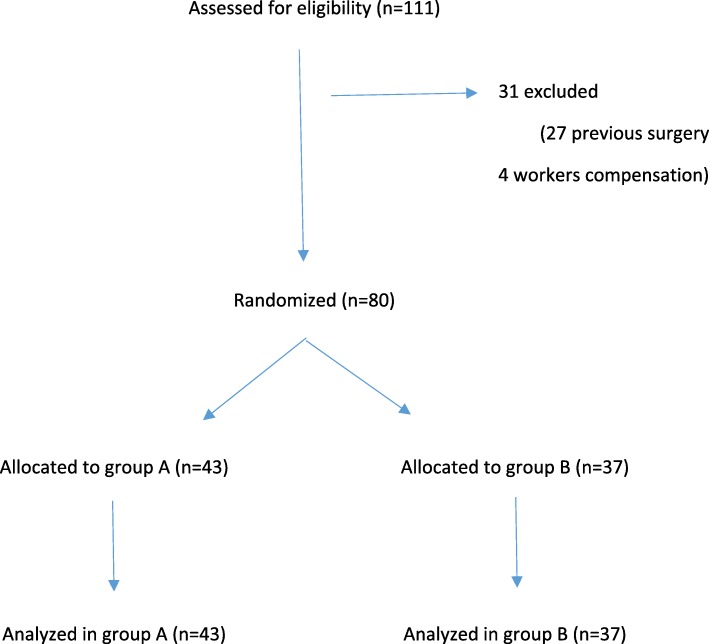
Flow diagram of the progress through the phases of a parallel-randomized trial of two groups

Patients were recruited from July to December 2014 in a single tertiary referral hospital. One hundred and eleven patients attended the Shoulder Unit for their first clinical visit during the recruitment period. Twenty-seven were excluded due to previous shoulder surgery and 4 were excluded as recipients of worker’s compensation. Eighty patients ultimately met the inclusion criteria (Fig. 1).

Randomization was performed with the aid of a computer-generated list with no restrictions. A nurse assigned new patients to either to group A or B, depending on the computer-generated list, before the clinical visit. The nurse asked the patients to complete a questionnaire before entering the office. The questionnaire included epidemiological data such as age and gender, clinical scenario A or B depending on the randomization, and the question to either accept or decline surgery. No information that could be used to identify the patient was recorded. The nurse administered the questionnaire on the first clinical visit after explaining that this was a study on patient decision-making to each patient, that it was anonymous and had no implication for treatment, and that involvement could be declined. The same doctor examined all the patients to minimize bias related to doctor attitude. Only oral information was given to both groups without any printed support. The key messages were that a torn cuff could be treated either conservatively or surgically.

The patients were randomly allocated to either group A (benefit) or group B (complication) and were asked to answer the following questions based on their assigned group:

Group A (benefit): Your doctor informs you that you have a rotator cuff tear and states that if he/she surgically repairs your cuff tear you will improve and that the cuff remains healed at the 2-year follow-up in 71% of the cases where surgery is done.

Would you choose surgery? Yes or No

Group B (complication): Your doctor informs you that you have a rotator cuff tear and that if he/she surgically repairs your cuff tear you will improve and that the cuff is torn again at 2-year follow-up in 29% of the cases where surgery is done.

Would you choose surgery? Yes or No

The data used to construct the question was obtained by arbitrarily choosing the study by Boileau et al. in which the authors declared that they obtained complete healing of the rotator cuff in 71% of the patients who underwent arthroscopic repair of full-thickness tears of the supraspinatus [17].

Age, gender, the shoulder affected, and the functional status assessed using the Constant score were also recorded [18]. The Constant score is a functional scale that includes subjective and objective data. Subjective data includes pain assessment (0–15 points) and daily living activities (0–20 points). Objective assessment includes range-of-motion (forward elevation, abduction, lateral rotation, and internal rotation) (0–40 points) and strength (0–25 points).

### Statistics

The power calculation for the sample size was based on an estimated reduction of 25% in the agreement to accept surgery, accepting an alpha risk of 0.05 and a beta risk inferior to 0.2. Forty patients were needed in each group to detect a significant difference between the two groups if the presence of the effect in group 1 was 0.6 and was 0.85 in group 2 using an ARCSINUS approximation test. Categorical variables are described with frequencies and percentages, and continuous variables are expressed as mean and standard deviations (SD). Comparisons of categorical variables between groups were assessed with the chi-square or Fisher exact tests, as appropriate. Quantitative variables were compared via the Mann-Whitney *U* test. *P* values of < 0.05 were considered statistically significant. Analyses were performed using the SPSS 18.0 software package (SPSS Inc., Chicago, IL, USA).

The study was approved by the Ethics Committee and all included patients provided signed informed consent.

## Results

There were 44 females and 36 males. The mean age was 50.8 years (ranging from 23 to 80). In 49 patients, the right shoulder was affected and the left shoulder in 31 patients. Forty-three patients were allocated to group A (benefit) and 37 to group B (complication). The mean age of the patients in group A was 50.8 years old and 54.5 years in group B. In group A, there were 29 females and 14 males while there were 25 females and 12 males in group B. The mean Constant score total was 62.3 for the patients included in group A and 58.2 for the patients in group B.

The overall results showed a significant difference (*P* = 0.000) between groups relative to their acceptance of surgery. Thirty-six out of the 43 (84%) of patients included in group A (benefit) accepted surgery compared to 17 out of the 37 (46%) in group B (complication).

Age, gender, the shoulder affected, and the functional status assessed by Constant score did not significantly differ between the patients accepting or refusing surgery (Table 1).

**Table 1:** Characteristics of patients accepting surgery compared to those refusing surgery

	Accepting surgery Yes	Accepting surgery No	*P* value
Question			
Benefit	36/43 (83.7%)	7/43 (16.3%)	
Side effect	17/37 (45.9%)	20/37 (54.1%)	0.000
Age	50.8	54.5	0.35
Gender			
Female	66.7%	33.3%	
Male	65.9%	34.1%	0.94
Laterality			
Right	67.3%	32.7%	
Left	64.5%	35.5%	0.79
Constant score	62.3	58.2	0.50
Pain	6.8	6.8	0.89
DLA	13.5	12.9	0.57
FE	8.4	7.9	0.43
ABD	8.3	7.8	0.46
LR	7.4	6.3	0.27
IR	7.2	7	0.66
Strength	10	8.9	0.45

*DLA* daily living activities, *FE* forward elevation, *ABD* abduction, *LR* lateral rotation, *IR* internal rotation

## Discussion

There is an increasing trend towards sharing the different treatment options with the patient and involving the patient in treatment decision-making [1–7]. This study demonstrates that doctors can significantly influence a patient’s decision depending on the content of the information provided. In rotator cuff disorders, most patients agree to receive surgery if the doctor presents information covering the basis of the benefits of surgery. On the other hand, most patients refuse to receive surgery if information is provided based on the negative outcomes of surgery, such as the cuff may be torn again at 2-year follow-up.

Not all physicians are in favor of involving patients in treatment decisions, especially less-experienced physicians. They are the ones who more frequently report barriers to involving patients [13]. Another commonly expressed barrier is the lack of time to share treatment decisions [13]. Nevertheless, it has been demonstrated that physicians are poor at assessing a patient’s preferences and that informed patients often tend to choose treatment strategies other than what the doctors suggest [11, 19].

From the patient’s perspective, not all want to be involved in treatment decision-making. Factors such as age, gender, educational level, urban or rural residence, and the severity of the illness play an important role in the decision [4, 9]. The Control preferences scale has been widely used to determine if the patient prefers an active, passive, or collaborative role in treatment decision [20]. Working on patient-reported outcomes rather than on functional scores and sharing this information with the patients as well as using it in the treatment decision-making process may be the path for the future [1].

There is no consensus about the best treatment strategy for rotator cuff disorders [21, 22]. While some authors recommend conservative treatment, others advocate for surgical repair of the torn cuff [8]. Among others, the re-tear rate after surgical treatment is a common complication that can impair outcomes [17]. Indications for rotator cuff repair have been defined from the physician’s perspective. These include age, gender, range-of-motion, weakness in forward elevation, tear size, and worker’s compensation status [8, 23, 24]. There is a lack of agreement among orthopedic surgeons relative to the indications for rotator cuff surgery. Surgeons who perform a higher volume of cuff repairs are more likely to agree to perform surgery [4]. From the patient’s perspective, psychological distress, depression, anxiety, and mental health seem to affect the outcome of rotator cuff treatments [24–28]. Moreover, whenever patient expectations were met after cuff repair, satisfaction, and quality of life improved [29, 30].

After all the aforementioned, it seems logical to involve the patient in the treatment decision-making for rotator cuff disorders but little attention has been paid to the way doctors inform patients so as to reach a decision. In the present study, it has been demonstrated that doctors can significantly influence a patient’s decision regarding rotator cuff surgery depending on the way they deliver the information. If information is presented based on the benefits of rotator cuff surgery, the majority of patients may accept surgery. Then again, most patients may refuse surgery if the information given focuses on the disadvantages of surgery. We cannot go about sharing information with patients and/or involving them in treatment decisions unless we are able to properly structure the information we provide and be sure that the information itself will not create a biased decision. It has been demonstrated, in urologic disorders, that the adverse effect profile of treatment is very important when deciding on the management of benign diseases, whereas treatment efficacy is more relevant in the management of life-threatening illness [10]. Full surgical information, including the characteristics of the arthroscopic technique, total length that the patient will need to be in-hospital until discharge, immobilization period, expected length of the rehabilitation process, and the time expected to fully recover and eventually to return to work, should be carefully explained to the patient.

In the present study, the level of pain or functional impairment, recorded using the Constant score, did not significantly differ between patients accepting or rejecting surgery. This suggests that the way that information is provided has a stronger influence on a patient’s treatment decision than the pain or functional impairment that the patient perceives. In the same manner, age, gender, and the shoulder affected did not significantly affect patients’ treatment decisions.

More research is needed on advising how to give information to patients and share treatment decision-making in rotator cuff disorders. Additionally, the development of decision-making support tools to help patients to make a decision for surgery or not in rotator cuff disorders should be considered as a potential solution.

The limitations of the study include the arbitrary selection of the Boileau et al. study to obtain the data to construct the question and that many factors can influence a patient’s decision. Despite these limitations, the randomized nature of the study provided a relatively equal distribution of various characteristics in both study groups that could potentially affect patient decisions. According to the sample size calculation, 40 patients were needed in each group. Eighty patients were included. Because of the randomization process, group A had 43 patients and group B had 37 patients, but the differences between both groups were wide enough to accept 37 patients in group B.

## Conclusions

The content of the information given to the patient influences their choice relative to treatment decision-making in rotator cuff surgery. Further research is needed to evaluate the effect of providing complete information to patients (specifying both sides, positive and negative) as well as the development of decision-making support tools that aid in choosing whether to do surgery or not for a rotator cuff disorder.

## Abbreviations

MRI: Magnetic resonance imaging
